# Supplementary figures and images for: Multi-modal investigation reveals pathogenic features of diverse DDX3X missense mutations
Source: PLoS Genet. 2025 Jan 21;21(1):e1011555. doi: 10.1371/journal.pgen.1011555 (PMC11771946; doi:10.1371/journal.pgen.1011555)

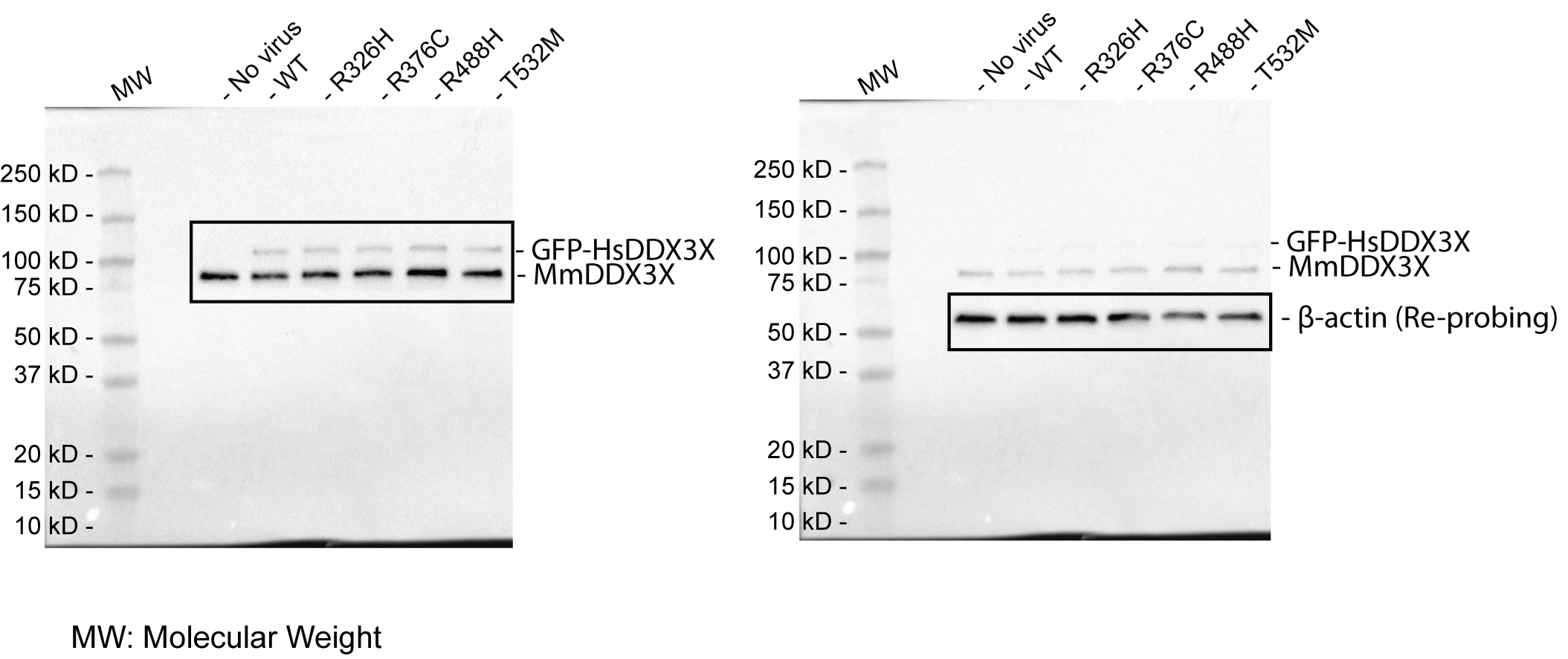

Supplement: S1 Data — (TIF) [file pgen.1011555.s006.tif]

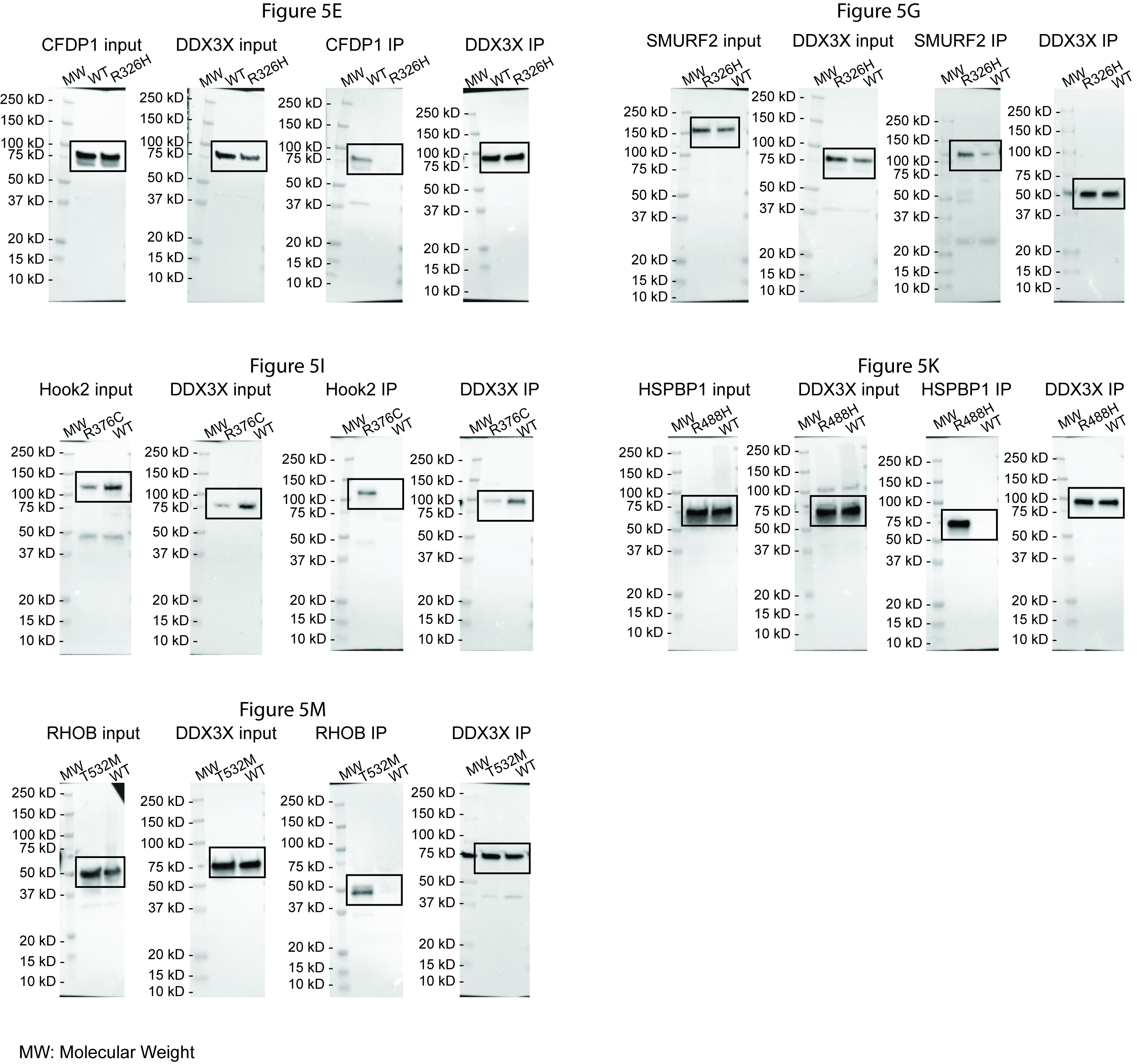

Supplement: S2 Data — (TIF) [file pgen.1011555.s007.tif]

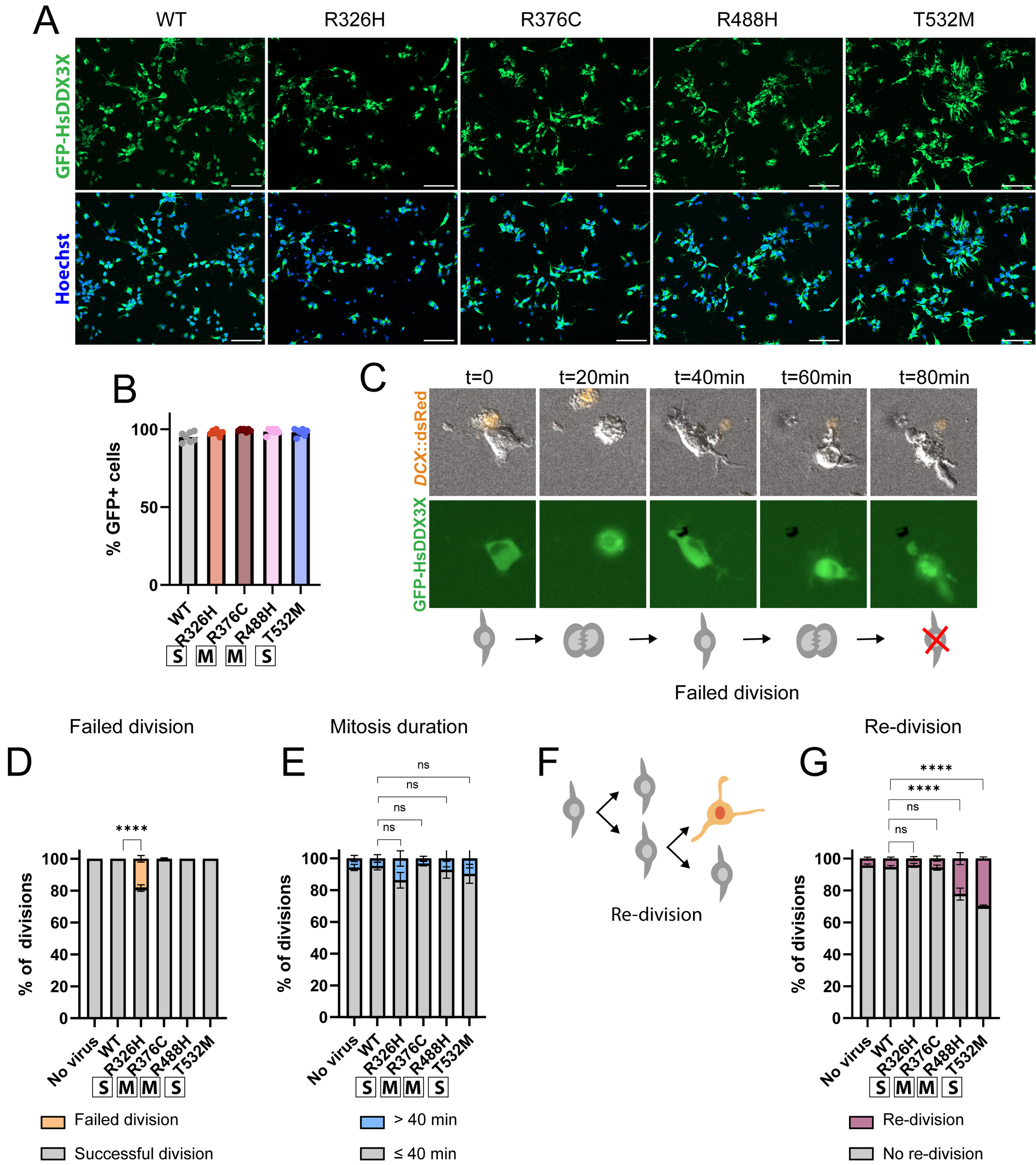

Supplement: S1 Fig — A) Low magnification representative images of mouse primary cultures 2 days after GFP-HsDDX3X lentiviral delivery. B) Quantification of transduction efficiency in A. Each dot represents a pooled primary culture. C) Snapshots of an unsuccessful mitosis and subsequent cell death. D) Quantification of failed divisions. E) Quantification of mitosis duration. F) Schematic example of a re-divisions. G) Quantifications of re-divisions. D, E, G) χ2 analysis with post-hoc Bonferroni. n = 3 live-imaging sessions and n = 3 litters. Scale bars: 10 μm. ****p < 0.0001; ns, not significant. Data are mean ± SD. “S”: severe; “M”: mild. (TIF) [file pgen.1011555.s008.tif]

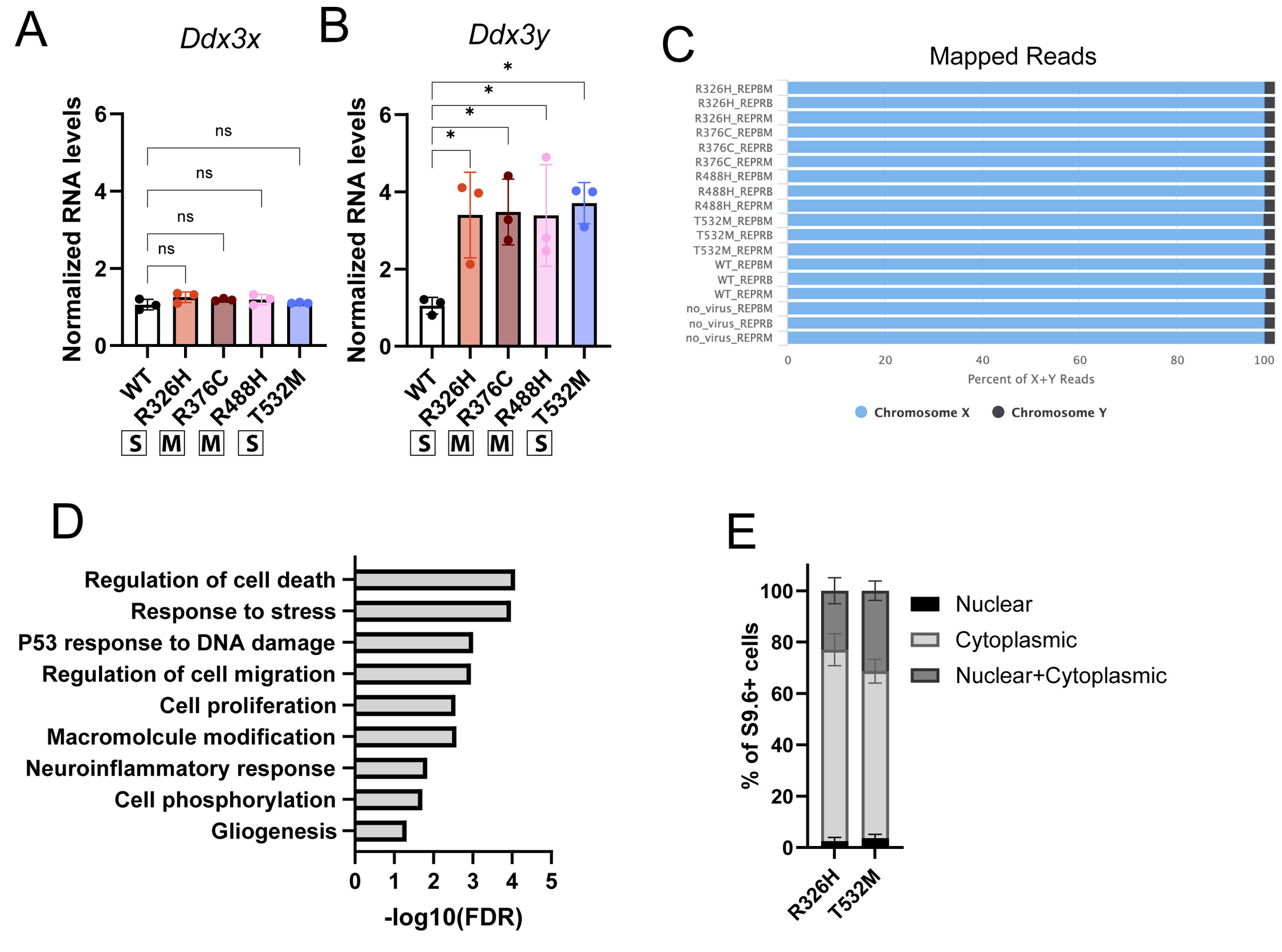

Supplement: S2 Fig — A, B) Ddx3x (A) and Ddx3y (B) levels from transcriptome datasets. C) Percentage of mapped reads from chromosome X and Y across all conditions and the three biological replicates of the transcriptome dataset. D) Gene ontology category relative to heatmap in Fig 3A. E) Quantification of sub cellular localization of S9.6+ cells. Two-way ANOVA. *p < 0.01; ns, not significant. Data are mean ± SD. “S”: severe; “M”: mild. (TIF) [file pgen.1011555.s009.tif]

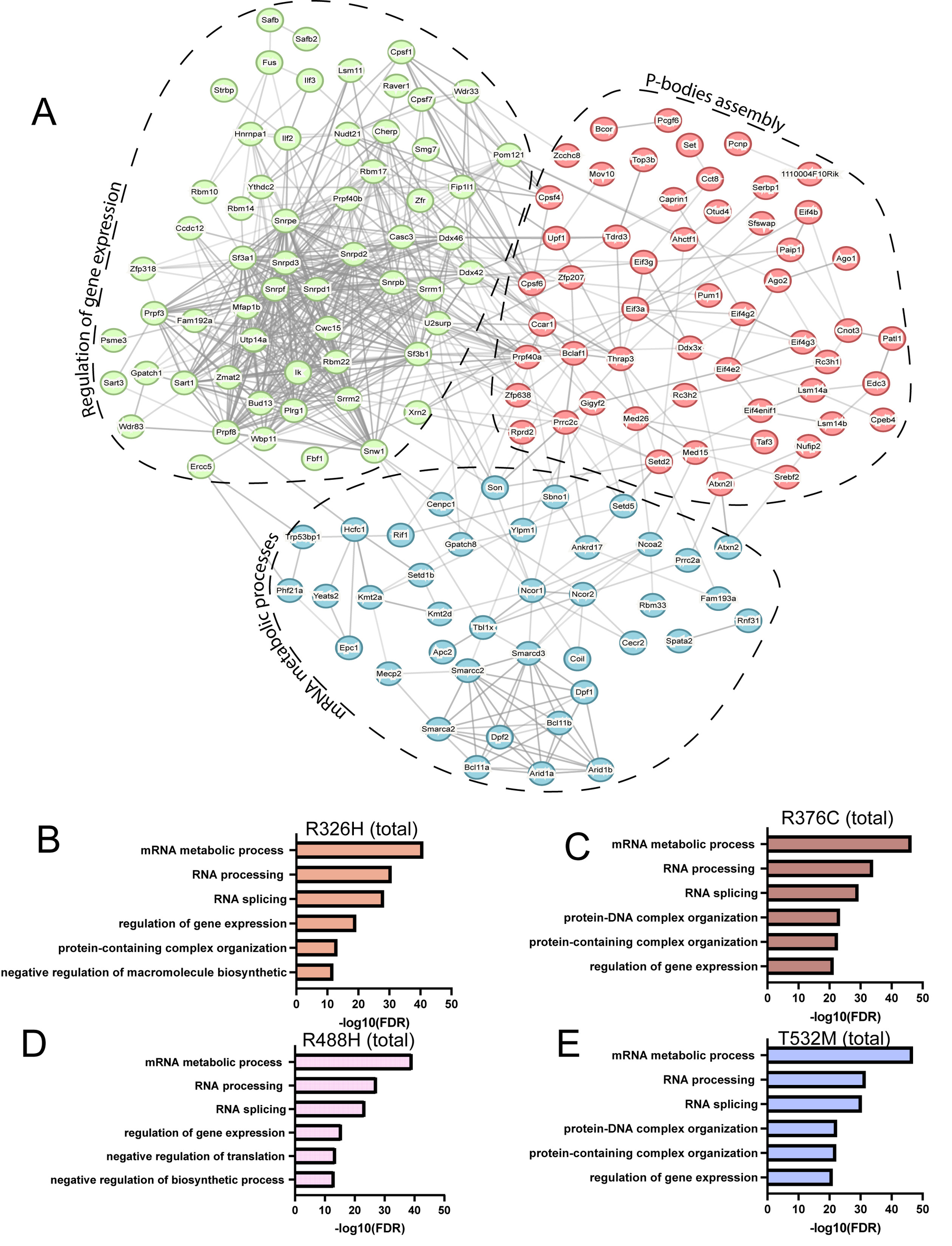

Supplement: S3 Fig — A) StringDB analysis of the 211 putative interactors shared between all experimental conditions. Nodes are divided according to GO categories. B, C, D, E) GO analysis of the significant interactors for R326H (B), R376C (C), R488H (D) and T532M (E) relative to the negative control. (TIF) [file pgen.1011555.s010.tif]

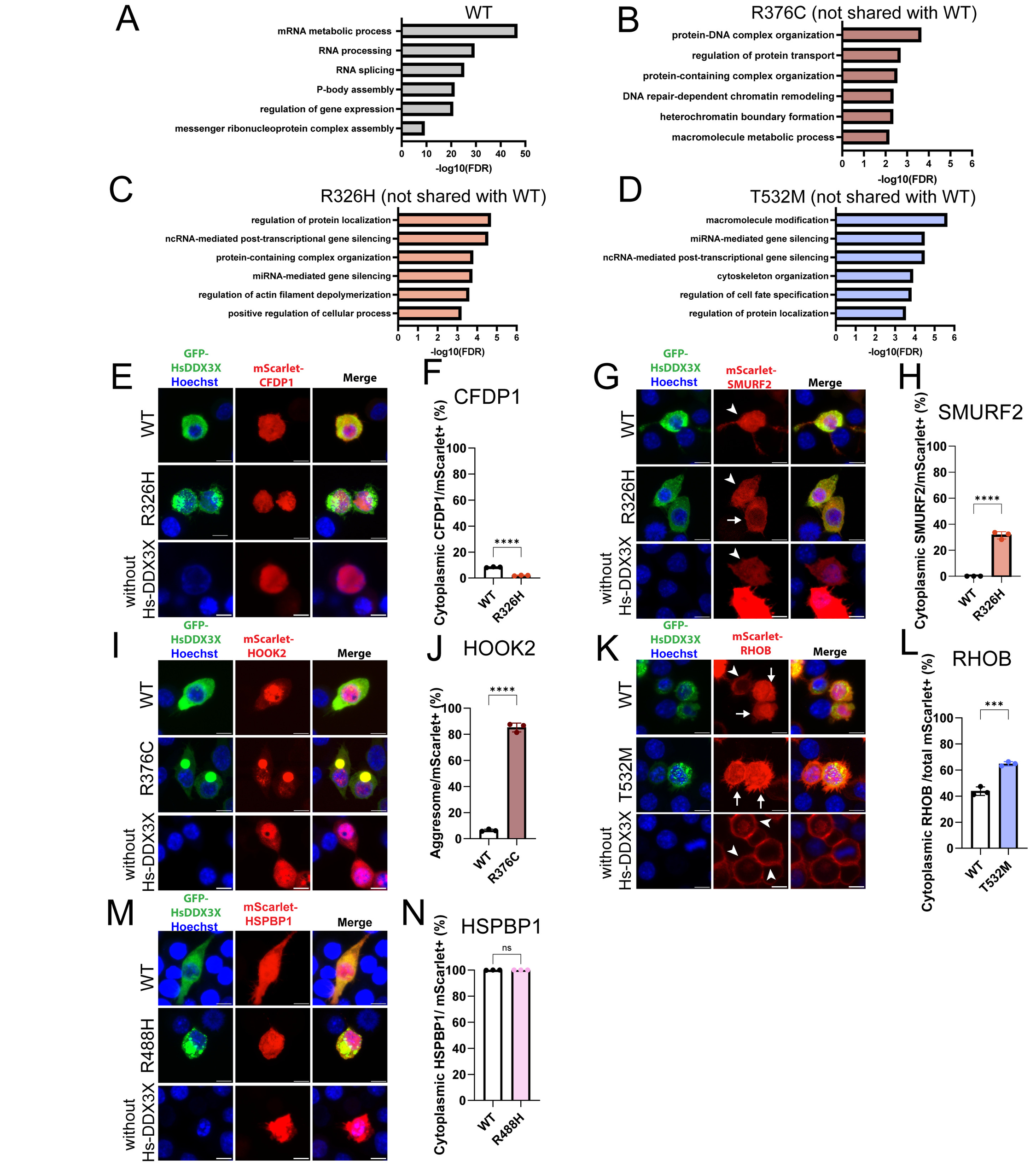

Supplement: S4 Fig — A–E) Gene Ontology analysis of the significant interactors for WT DDX3X (A). and those not shared with DDX3XWT for R326H (B), R376C (C), T532M (D). E, F) Representative images of N2A cells transfected with GFP-HsDDX3X (green) and mScarlet-CFDP1 (red) (E) and quantifications (F). G, H) N2A cells transfected with GFP-HsDDX3X (green) and mScarlet-SMURF2 (red) (G) and quantifications (H). Arrowheads point to example of whole cell localization, arrows to cytoplasmic localization. I, J) N2A cells transfected with GFP-HsDDX3X (green) and mScarlet-HOOK2 (red) (I) and quantifications (J). K, L) N2A cells transfected with GFP-HsDDX3X (green) and mScarlet-RHOB (red) (K) and quantifications (L). Arrowheads point to example of membrane localization, arrows to cytoplasmic localization. M, N) N2A cells transfected with GFP-HsDDX3X (green) and mScarlet-HSPBP1 (red) (M) and quantifications (N). F, H, J, L, N) each dot represents an independent transfection. Scale bars: 10 μm. Unpaired t-test *p < 0.01; **p < 0.001; ***p < 0.0001; ****p < 0.0001. Data are mean ± SD. (TIF) [file pgen.1011555.s011.tif]

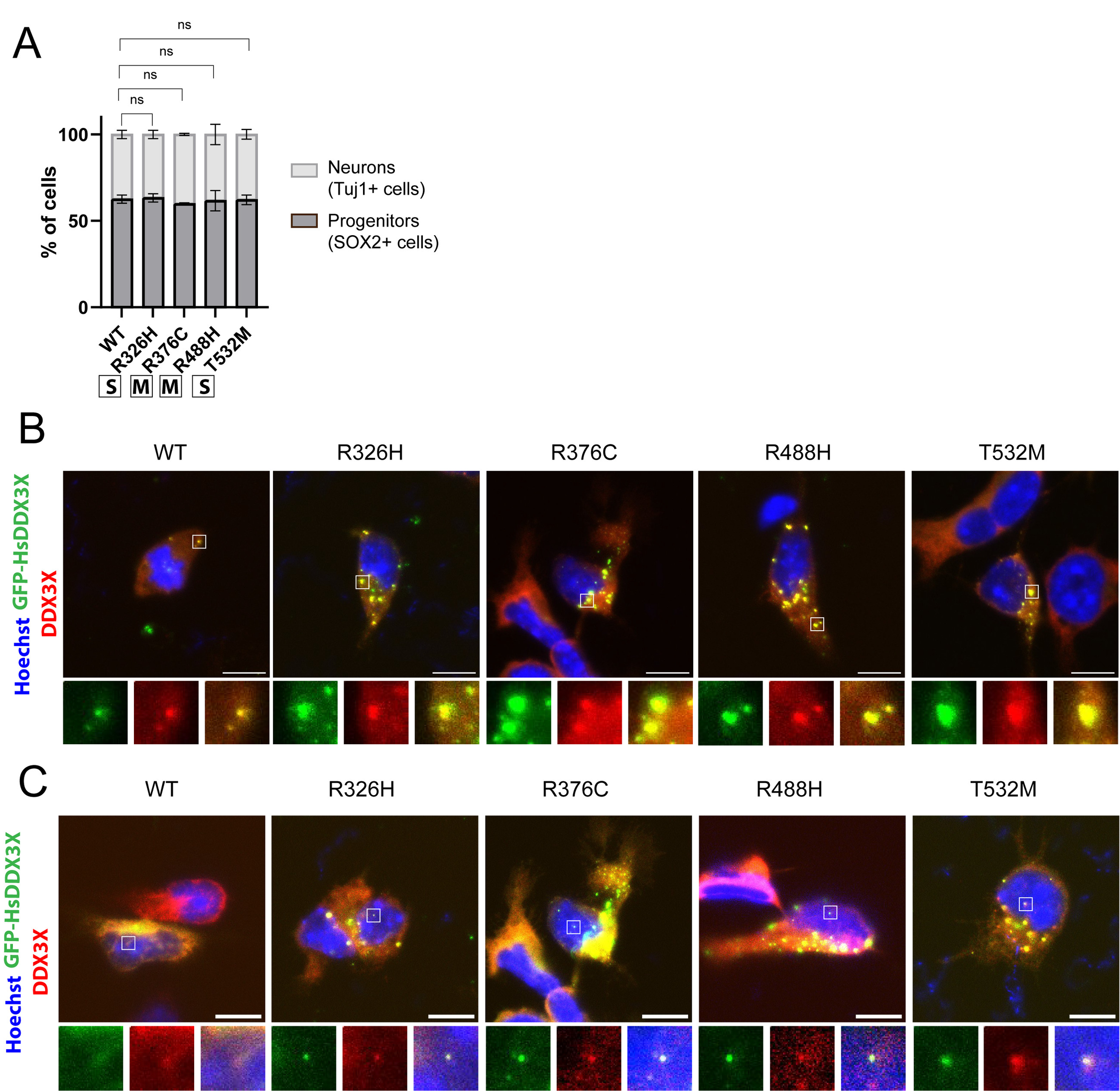

Supplement: S5 Fig — A) Quantification of total cell composition (SOX2 and Tuj1+ cells) of primary cells after 2 days of lentiviral transduction. B) Example of staining with anti-DDX3X (red) and GFP (green). Inserts show overlap with GFP-HsDDX3X granules. C) Example of staining with anti-DDX3X (red) and GFP (green). Inserts show overlap in nuclear puncta of DDX3X. Scale bars: 10 μm. Two-way ANOVA ns, not significant. Data are mean ± SD. “S”: severe; “M”: mild. (TIF) [file pgen.1011555.s012.tif]
