# Supplementary material for: Multi-modal investigation reveals pathogenic features of diverse DDX3X missense mutations
Source: PLoS Genet. 2025 Jan 21;21(1):e1011555. doi: 10.1371/journal.pgen.1011555 (PMC11771946; doi:10.1371/journal.pgen.1011555)
Supplement: Attachment — Submitted filename: Mosti et al response to reviewers.pdf [file pgen.1011555.s013.pdf]

Response to Reviewers, PGENETICS-D-24-01170

Reviewer #1:

The manuscript by Mosti et al. entitled “Multi-modal investigation reveals pathogenic features of diverse DDX3X missense mutations” characterized four clinical missense mutations in DDX3X that cause mild to severe anatomical defects and behavioral phenotypes. Authors leveraged a primary mouse neural progenitor cell model to study the impact by these mutations on cell fate during early development. They first optimized the expression level of GFP-tagged human DDX3X in mouse, with the missense mutant proteins expressed at ~30% of the endogenous mouse DDX3X level. Under these conditions authors showed that these mutations differentially impact cell fate and survival, with the clinically severe mutations cause more proliferation, less neurogenic cell division, and more neuronal apoptosis. They also showed transcriptomic upregulation of DNA damage response pathways in cells expressing the mutants. They then investigated DNA damage levels by immuno-staining for DNA double strand breaks and R-loop. They showed high levels of gH2A.X and cytoplasmic R-loop associated with the severe mutations in Tuj1+ neurons, leading to the hypothesis that R-loop-induced DNA damage underlies neuronal apoptosis. Finally, they analyzed the protein interactome of DDX3X mutants as well as stress granule formation in cell expressing the mutants. This is the first study to demonstrate that DDX3X missense mutations impair neurogenesis and neuronal survival. The discovery of increased DNA damage and R-loop formation in cell expressing mutant DDX3X, and particularly those clinically severe mutants, bears importance on the etiological basis for DDX3X Syndrome and associated neurodevelopmental disorders. The experimental model is well chosen, the data are clearly presented, and the manuscript is well written.

We thank the reviewer for the excellent summary and their helpful comments.

I only have one **major point** and a couple of minor concerns/suggestions for the authors. The major point regards the observation of increased R-loop level mainly in the cytoplasm of cells expressing the mutants. The authors hypothesized that (nuclear) R-loops translocate into the cytoplasm, causing accumulation of stress granules, ultimately leading to apoptosis. This translocation hypothesis remains untested and may not be the parsimonious explanation. Because the S9.6 antibody is known to react with dsRNA in addition to the RNA:DNA hybrid, cytoplasmic S9.6 signals are largely attributable to dsRNA. Meanwhile, the nuclear S9.6 signals were not quantified. Based on the legend of Fig. 4, authors quantified the percentage of cells showing positive signals for S9.6 among GFP+ cells, instead of quantifying the level of S9.6 (or other DNA damage markers) signals. This practice might overlook those S9.6 signals in the nucleus, which are more likely due to co-transcriptional R-loop formation. Therefore, I suggest that the authors re-quantify nuclear S9.6 signals using Hoechst dye to define the nuclear perimeter and ask if mutant DDX3X cause increased nuclear R-loop formation as well. If feasible, it would be great to repeat the immunostaining for R-loop with and without RNase III treatment to remove dsRNA and then analyze nuclear S9.6 signals and stress granule formation in the cytoplasm. I feel these experiments would be crucial for demonstrating co-transcriptional R-loop formation causing DNA double strand breaks due to DDX3X mutations.

We agree with the reviewer that localization analysis for the R-loop signal is helpful to support our conclusions. As suggested, we quantified the signal intensity of S9.6 antibody in the nuclei, by using the Hoechst signal to generate a nuclear mask in Fiji. The results show significant S9.6 enrichment for DDX3X^{R326H} and DDX3X^{T532M} in the nucleus, a trend that is similar to what we reported in Figure 4G. While analyzing, we noticed that only a subset of cells positive for S9.6 showed nuclear signal, so we also quantified the subcellular distribution of the S9.6 signal in DDX3X^{R326H} and DDX3X^{T532M} expressing cells (Cytoplasmic, only Nuclear, or Nuclear and Cytoplasmic). ~70% of S9.6+ signal was cytoplasmic, ~25% was nuclear and cytoplasmic, and >4% was only nuclear. Because the nuclear signal was only a very small subset of cells, we have added only these data (on the right, below) to Sup. Fig. 2E. We discuss this point in the results (p. 11-12).

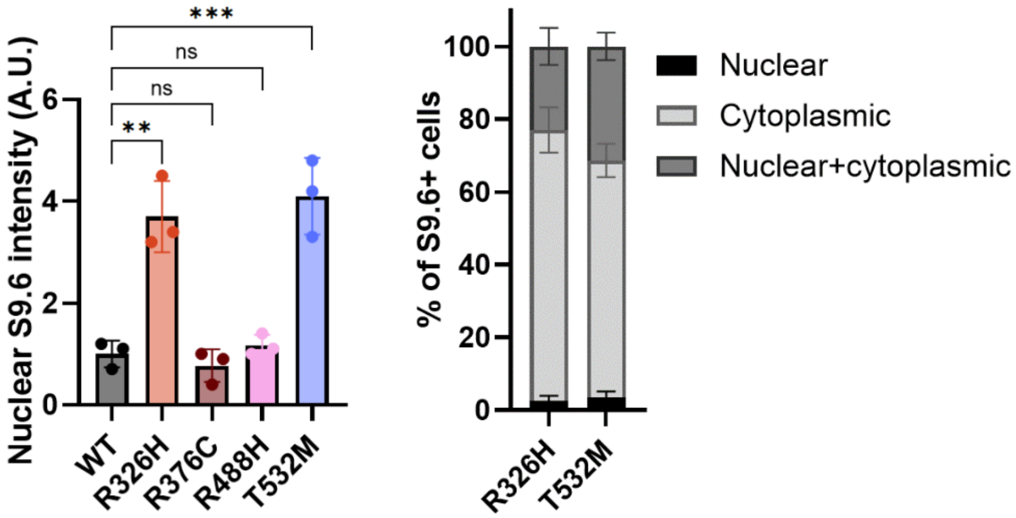

Minor points:

1. The statement "Due to its location on the X chromosome, DDX3X syndrome predominantly affects females, although a growing number of males have been identified" is a bit mis-leading. Typically an X-linked mutation would be considered to affect males predominantly instead. I thought the reason why DDX3X mutations show a female-bias is because hemizygous mutations in males are likely lethal.

We agree with the reviewer's suggestion and have modified the wording in the introduction (p. 4).

2. The DNA damage response pathway markers ATM, ATR, DNA-PK, etc. all showed up-regulation in cells expressing the mutant DDX3X. These markers suggest that increased DNA damage might be due to DNA replication (ATR, DNA-PK) defects, in addition to transcription-associated DNA damage. This might be an important point given the observation of increased proliferation in the mutant cells.

This is a good suggestion, and we added this point to the results (p. 10).

3. Related to the point #2, might it be that the DNA damage in neurons is due to re-entry of the cell cycle? It would be nice to see some in-depth discussion on the specific DNA damage response.

We agree with the reviewer and we include a new paragraph in the discussion (p.18-19).

Reviewer #2:

This was a well written paper addressing an important question of the consequences of patient missense mutations in DDX3X. They used an NPC model system, and evaluated the consequences of 4 mutations in a very careful way, with a range of different approaches. They carefully thought about how to titrate the lenti to express the DDX3X variants and near endogenous levels. They confirmed their level of overexpression had no transcriptional effects. I like the use of the S and the M in the figures to keep track of which was severe and which was mild. They had nice follow up of initial transcriptional discoveries with cell biological approaches to support their conclusions. On the whole, it was an important and well executed paper that should merit publication in Plos Genetics, and they mostly support the claims they made. I have only a few minor points where I thought they needed a bit more support, and a few minor clarifying points.

Claims:

1) Diverse DDX3X missense mutations disrturb neural dev. through distinct cellular and molecular mechanisms, comparing two missense from severe patients and two from mild
2) Severe mutations impair neurogenesis and cause cell death, while mild mutations have a mild effect.
3) Different mutations have different interactors by proximity labeling, with severe ones sharing more neighbors.
4) Transcriptomics identifies pathways, including DNA damage response, and clinically severe mutations show DNA damage in neurons.
5) These may be due to DNA:RNA hybrids and formation of stress granules, pointing to a new mechanism of disease.

I found they mostly supported the claims, though it was not the clear to me the stress granules were part of the disease pathway (they could be a correlate), and I though they could strengthen the analysis for #3, as described below.

We thank the reviewer for the excellent summary and their helpful comments.

Suggestions:
A. It is a bit strange that DDX3X is having such a nuclear consequence for a cytoplasmic protein, and this doesn't readily fit into their model. Could the assumption that DDX3X is cytoplasmic (as mentioned in the intro) be an oversimplification, and it is also in the nucleus at low levels or at certain puncta? The authors could consider some careful Immunofluorescent quantification of DDX3X (both their tagged versions, as well as endogenous) in the nucleus, to see if there are trace amounts. This may be possible from reanalysis of their existing collection of images. Or potentially this could be done with nuclear vs. cytoplasmic fractionation followed by Western, or perhaps over-ambitiously, Cut N Run to see if DDX3X associates with parts of DNA where R-loops are formed. Potentially their proteomics might tell them something too (is it associating with nuclear or cytoplasmic proteins). Overall, I recommend some limited additional experimentation exploring if its sub-cellular localization can support (or at least not refute) their model. If DDX3X does not colocalize with the Rloops staining, DSB, etc, in nucleus, this suggests that the impact of DDX3X on these phenomena is indirect rather than direct (perhaps disrupting translation of nuclear helicases or BRAC1A, for example).

We agree with the reviewers that our description of DDX3X localization to cytoplasm was oversimplified. While DDX3X is mainly cytoplasmic, data from our previous work utilizing a different anti-DDX3X antibody shows it also has some nuclear localization (Lennox et al., 2020). We have modified the introduction to indicate this (p.5).Further, as suggested by the reviewer, we include new images to show GFP-DDX3X and endogenous DDX3X punctae in the nucleus(Sup. Figure 5C). We also have added this to the results (p.14).

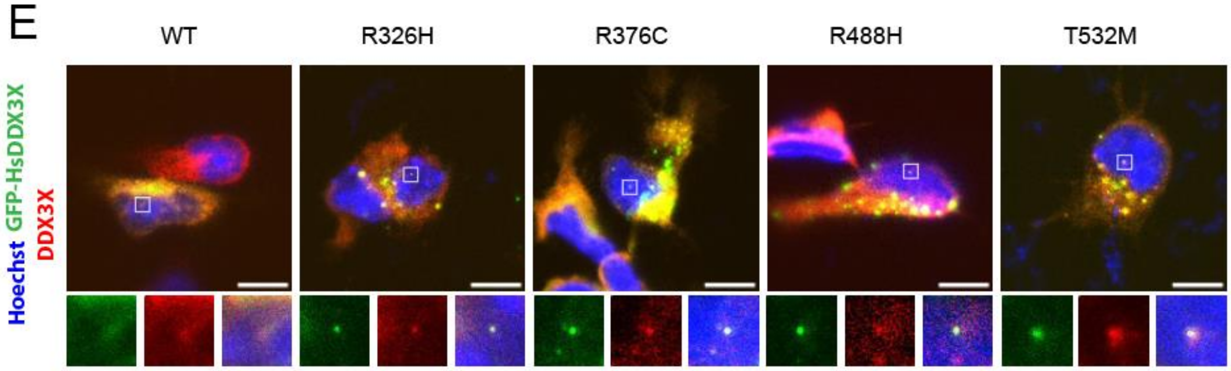

B. In the proteomics, addition to comparing each tagged protein to untagged Turbo ID, if they want to conclude that mutants are different from WT DDX3X, they should also compare them directly and statistically to WT DDX3X. Of course, they should probably limit this analysis to the those proteins are significantly enriched in the first analysis of comparing the DDX3Xs untagged Turbo ID. I would recommend this as additional columns in existing sup sheets, as well as perhaps an additional figure panel like 5B, but with the log FC and p-value coded as WT vs Mutant. I think this added analysis is important because their primary conclusions for claim #3 are about the difference between WT and mutant, and thus their should be a statistical analysis that directly asks about the difference between WT and mutants. Currently it appears the analysis compares each of these to a third, untagged, samples instead. Such a direct comparison would improve their support of claim #3.

This is a great suggestion. While our initial submission discussed this comparison between WT DDX3X and specific DDX3X mutants (Figure 5C, Sup. Fig. 4A-D), the specific values of this analysis were missing in the supplemental table. We have added these columns to Table S4.

C. Do the authors have any samples from their historical DDX3X LOF mutants to look at marks of DNA damage and R loop formation? That would increase the impact of the pathways discovered here by showing some shared mechanism with the LOF perhaps. The negative results is also interesting, and would argue strongly LOF and severe missense are different diseases.

We appreciate the reviewer's question. We do not expect to observe DNA damage or R-loops in our Ddx3x LoF mouse model for multiple reasons:

- The LoF model is heterozygous leading to around a 50% loss of *Ddx3x*. Previous work has shown that a 70-80% reduction of *DDX3X* expression is needed to observe any DNA damage (Randolph et al., 2024).
- Acute manipulation of *Ddx3x* levels might be necessary to observe DNA damage accumulation and R-loops cytoplasmatic translocation. Indeed, in cancer cells levels of DNA damage induced by 6hrs of *DDX3X* downregulations were almost halved after 24hrs (Randolph et al., 2024). This might suggest a partial mechanism of cell recovery. Our mouse LoF model induces loss of *Ddx3x* is days prior to analysis, suggesting a most likely absence of DNA damage markers.
- In agreement with the previous points, Ddx3x LoF heterozygous do not present cell death in the cortex.

Finally, it is not necessary to support their claims, but it might be a cool experiment that further supports their model to overexpress RNASE H, or use some other treatment that resolves R loops and determine if this improves their severe mutants in terms of DSBs and apoptosis (e.g, https://www.nature.com/articles/s41467-020-18306-x), or cytoplasmic R-loops specifically, if they think these are mediating the apoptosis, as was shown in this recent study: https://www.nature.com/articles/s41586-022-05545-9. Likewise, do the authors think it is the stress granules that matter for death, or activation of IRF3 signaling as in https://www.nature.com/articles/s41586-022-05545-9? This could be at least worth some discussion, if not some Westerns similar to the prior work.

We thank the reviewer for the suggestion. We expanded our discussion points on R-loops, inflammation and apoptosis (p. 19).

Minor:

In figure 8, is the little purple-blue shape meant to be DDX3X? Maybe add that to the key.

We agree this was confusing and have added it to the legend.

Methods: how the statistical comparison of samples was done for proteomics is missing.

We apologize for this omission, the methods have been updated accordingly (p. 28).

Supplemental tables could each use legends or README sheets to explain what the column headers mean. This will make the data reusable for others.

We thank the reviewer for this suggestion. "READ ME" tabs have been added to supplementary tables 2-4.

How do they think about X inactivation. Is that relevant here? Or does this escape X-inactivation? If so, it might be worth mentioning, at least parenthetically.

Thanks for the question. *DDX3X* is one of the few genes that escape X-inactivation in human cells. We have added this to the discussion (p. 17).

2F,G. What are the two factors in the two way anova? I only see one factor, unless sex was the other? I have a similar question about other figures.

We used two-way Anova in all graphs in which we were performing multiple comparisons across >2 conditions. Post-hoc Sidak analysis was performed for pairwise-analysis and these are the statistics which are shown in figures. This is described in the methods.

DDX3Y western, to see if it is upregulated at protein level, would also be interesting (though could be tangential, and not required). But, perhaps they could at least discuss why increased DDX3Y does not rescue DDX3X deficiency?

This is an interesting question. Unfortunately, the DDX3X and DDX3Y sequences are too similar for any antibody to clearly distinguish them. We added a new paragraph to the discussion to address regarding the relationship between DDX3 paralogs (p. 17).

Line 254: for a second I thought they might have had a typo to mean ‘nuclear accumulation of R loops’ rather than cytoplasmic? So it was initially a bit confusing . T532M looks nuclear, and R326H looks cytoplasmic. But, that is because I was not aware of the very recent literature on cytoplasmic R loops. It might be worth mentioning those citations right away, since cytoplasmic R loops are a recently reported phenomena.

We apologize for the confusion. We did mean “cytoplasmic” accumulation. We have added these references as suggested to the results. We did not observe any differences between T532M and R326H in terms of R loop localization (please see response 1 to Reviewer 1). Thus, we include a new representative Figure 4C for T532M to reduce the confusion.

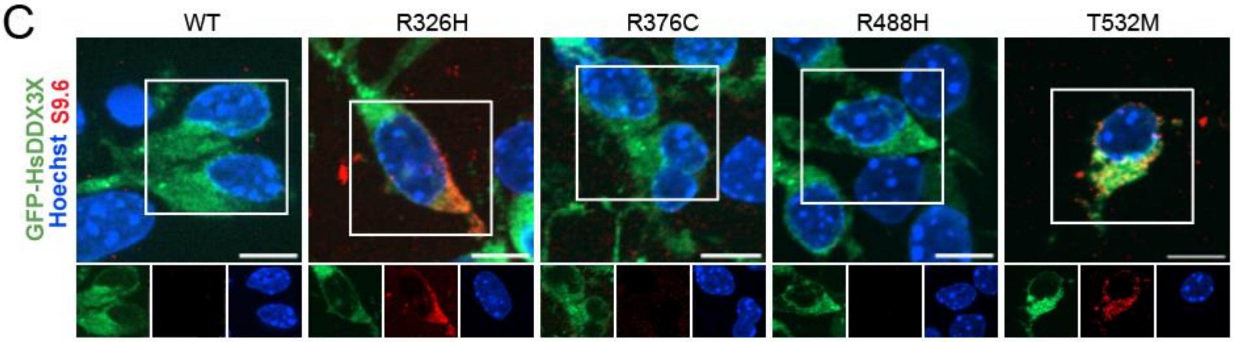

Reviewer #3:
In this study, the Authors characterise the cellular and molecular mechanisms underlying clinically severe and mild mutations by integrating transcriptomics, proteomics, and live imaging. The study uses primary mouse neural progenitors to reveal how these mutations differentially affect neurogenesis, neuronal survival, and key pathways such as RNA metabolism and DNA damage response.
Suggested Additions:

1. Discussion Expansion:
The discussion should delve deeper into the role of R-loops in inducing neuroinflammation. Highlight their capacity to trigger immune responses, emphasizing the link between unresolved DNA:RNA hybrids and chronic inflammation in neural tissues. This could include potential therapeutic approaches targeting R-loop resolution to mitigate inflammation and its downstream effects.

We thank the reviewer for the suggestion and have added a new paragraph to the discussion (p. 19).

2. Conclusion Expansion:
The conclusion should outline how these findings can inform clinical strategies, particularly the development of targeted therapies for severe DDX3X mutations. Highlight opportunities for personalised medicine based on molecular phenotyping of specific mutations.

We thank the reviewer for the suggestion and have added a new paragraph to the discussion (p. 20).
